# The attitudes of healthy children and researchers towards the challenges of involving children in research: an exploratory study

**DOI:** 10.1186/s40900-021-00263-4

**Published:** 2021-05-14

**Authors:** Laura Postma, Malou L. Luchtenberg, A. A. Eduard Verhagen, Els L. M. Maeckelberghe

**Affiliations:** 1 Wenckebach Institute for Education and Training, University of Groningen, University Medical Center Groningen, Hanzeplein 1, 9713 GZ Groningen, the Netherlands; 2 University of Groningen, University Medical Center Groningen, Beatrix Children’s Hospital, Groningen, the Netherlands

**Keywords:** Patient and public involvement, PPI, Children, Child-inclusive research, Healthy children, Co-researchers, Paediatric research, Medical research, Medical researchers

## Abstract

**Background:**

A growing trend in research is to involve co-researchers. It is referred to as Patient and Public Involvement (PPI) and comprises three groups: the patients, the public, and the researchers. Like in adult public involvement, healthy children can also be considered as ‘the public’. Paediatric patients and researchers experienced in conducting child-inclusive research are often asked about their attitudes towards the challenges they encounter. This is not the case for healthy children and researchers without such experience. Our aim was to investigate the attitudes of these children and researchers towards the challenges encountered during child-inclusive research.

**Methods:**

This was an exploratory study. We interviewed healthy children and adult researchers without prior experience in child-inclusive research. We recruited the children through a foundation for young researchers and the adult researchers from two hospitals, both in Groningen, the Netherlands. We audio recorded the interviews, and they were transcribed verbatim. We analysed the data using qualitative content analysis.

**Results:**

We interviewed five adult researchers and seven healthy children, aged 9 to 14 years. Both groups thought that it was best to involve children in paediatric research from as early a stage as possible. The children assumed that no prior training would be needed because they had already been trained at school. The researchers’ attitudes varied regarding training children beforehand. Both groups thought that researchers did not need prior training on how to involve children if they worked with children on a daily basis. The children felt that recognition and a modest financial reward was appropriate. Adult researchers were cautious about rewarding the children. They feared it might render the children less intrinsically motivated.

**Conclusion:**

Our study indicated that young and adult researchers have clear attitudes towards the challenges encountered during child-inclusive research. Young researchers could help adult researchers to find solutions to these challenges, even if they have no prior experience in child-inclusive research. Adult researchers who acknowledge the importance of child-inclusive research represent a significant step towards meaningful involvement of children. Our results imply that children could be involved in the decision-making process concerning the challenges encountered in child-inclusive research.

## Plain English summary

A growing trend in research is to involve co-researchers. It is called Patient and Public Involvement (PPI) and includes three groups: the patients, the public, and the researchers. In child-inclusive research the patients are children with a medical condition while the public is represented by healthy children. Generally, the attitudes of patients and researchers are heard, while the public is often unheard. We aimed to give a voice to healthy children and researchers without prior experience in child-inclusive research to determine their attitudes towards the challenges encountered.

We interviewed seven healthy children and five researchers without prior experience in child-inclusive research. Both groups thought that it is best to involve children in research from as early a stage as possible. The children saw no added value in training children to be involved in child-inclusive research. The attitudes of researchers varied on this point. Both groups thought that researchers did not require prior training on how to involve children provided they already worked with children on a day-to-day basis. Children thought a modest financial reward would be appropriate, while researchers were cautious about giving a reward.

Our study indicated that both groups had clear attitudes about the challenges encountered during child-inclusive research. Young researchers could help adult researchers to find solutions to these challenges, even if they have no prior experience in child-inclusive research. Our results imply that children could be involved in the decision-making process concerning the challenges encountered in child-inclusive research.

## Introduction

A growing trend in research is to involve co-researchers or advisors. This is referred to as Patient and Public Involvement (PPI) and defined as research conducted ‘with’ or ‘by’ members of the public rather than ‘to’, ‘about’, or ‘for’ them [1]. Such involvement extends their roles beyond that of research subject or participant. Funding agencies encourage researchers to involve patients for various reasons: patients may help researchers to understand their research results better, patients may raise research questions relevant to them, or because patients may provide insights or expertise lacking in other members of the research team [2].

Patient and public involvement comprises three groups: the patients, the public, and the researchers. As a rule, patient involvement and public involvement are grouped under a common heading [2]. Frederiksson and Tritter, however, highlight the distinction between patient involvement and public involvement. Typically, patients are expected to have a vested interest related to their care, whereas the public is expected to provide a disinterested perspective [3]. To complement the attitudes of the patients, the public could be involved in research to add a level of impartiality or independence [2].

In the early 1990s, a new era emerged in children’s rights. Article 12 of the United Nations Convention on the Rights of the Child (UNCRC) states that children have the right to express their views on all matters affecting them and that those views are given due weight. The UNCRC further states that children should have a say in research if it affects them [4]. Recently, children have been involved in child-inclusive research more often. Wilson and colleagues found that out of 187 studies involving children in research, 98 (52%) were published between 2016 and 2020 [5]. In our article we refer to the involvement of children in research as child-inclusive research.

Child-inclusive research is believed to have a positive impact on children, on researchers, and on research itself. Children gain self-confidence, their understanding of research improves [6, 7], and it offers them the experience that their impact on research matters [7–9]. Researchers obtain a greater understanding of the children’s needs, which enables them to adjust the research accordingly [10], and their respect for children increases [6]. The quality of research improves, prioritizing research questions is based more on relevance and importance to children than was the case previously, and the children’s perspective is added to the data analysis, which is unique [7].

Calls appeared in the literature for research groups to share their experiences regarding the lessons learnt and the challenges encountered. Their aim was to thus establish best practice guidelines for meaningful child-inclusive research [11–14]. Hoven and colleagues interviewed paediatric patients about their experiences as research partners [15]. Researchers experienced in child-inclusive research have regularly reported on the challenges they encountered [12, 16–18]. These challenges include the requirement of comprehensive training programs, concerns regarding power imbalances, protection of children’s needs [18], development of methodologies encouraging children to speak for themselves and to heed their advice, how much do children contribute, and how can they be rewarded best [12].

It is noteworthy that paediatric patients are heard about their attitudes towards the challenges encountered during child-inclusive research more often than are healthy children. Like in adult public involvement, healthy children could be regarded as ‘the public’, and they should complement the attitudes of paediatric patients. Additionally, paediatric patients may have experienced research during their time in hospital. It could be that these children know more about research than healthy children and may therefore have different attitudes towards the challenges encountered. Paediatric patients, for example, might be more willing to be involved in research without receiving a reward because they are the ones who benefit from it directly.

Researchers with experience in child-inclusive research are asked about their attitudes towards challenges in child-inclusive research more often than researchers without such experience. Nevertheless, it is important to be aware of the attitudes of both groups of researchers. Possibly, researchers with experience in child-inclusive research are more positive towards the challenges encountered during such research than researchers without this experience. To increase child-inclusive research, we believe that it is important to also give a voice to researchers without experience in such research and to thus learn about their attitudes.

The aim of this exploratory study was to investigate the attitudes of healthy children and researchers without experience in child-inclusive research towards the challenges encountered during child-inclusive research.

## Methods

This was an exploratory qualitative study using semi-structured interviews, conducted groupwise in case of the young researchers and individually in case of the adult researchers. We endeavoured to collect data from two less frequently researched groups: healthy children without prior experience in child-inclusive research, whom we referred to as the young researchers (YRs) and adult researchers (ARs) without prior experience in child-inclusive research. We deliberately chose to include children rather than parents. Although we are aware that parents may influence their children’s decisions, we did not elaborate on this issue in this study.

### Research team

The research team consisted of a research trainee (LP), a medical doctor (ML), an ethicist (EM), and a paediatrician (EV). All were experienced researchers in the field of paediatric qualitative interview studies.

### Recruitment and sampling

We included healthy children, aged 8 to 18-year-old, who displayed an interest in research or who had some research experience. We defined experience as ‘children with some research knowledge and research skills’, but strictly without any prior experience in child-inclusive research. We deliberately included YRs with an interest in or with some experience in research in order to compare their perspectives with those of ARs. We made an important distinction between child participation and child involvement in research. We formulated the inclusion criteria for ARs in line with the inclusion criteria for YRs. Adult researchers were included if they were experienced paediatric researchers with at least one publication. They should have had no prior experience in child-inclusive research (Table 1). We requited the YRs through *Stichting De Jonge Onderzoekers Groningen* [a foundation for young researchers in Groningen, the Netherlands]. This foundation offers children the opportunity to develop their interests in technology and science. Thus, our YRs represented a purposeful and convenience sample. We recruited the ARs through a university medical centre and a general hospital also located in the north of the Netherlands. As this was a small-scale, exploratory qualitative study we did not aim to reach data saturation. Nevertheless, during a 6- month period, we endeavoured to include as many participants as possible.

**Table 1:** Inclusion criteria

**Inclusion criteria**	
**Children**	
Children aged 8 to 18 years	
Children with some experience in participating in research / interest in research (participation)	
Children with no previous experience in child-inclusive research (involvement)	
**Researchers**	
Experienced paediatric researchers with at least one publication	
The publication or publications should address a paediatric topic without involving child-inclusive research	

### Informed consent

During one of the meetings at the *Jonge Onderzoekers Groningen* foundation, we informed the YRs about the research and we asked them to participate in the interviews. We handed out the information sheets and informed consent forms. In addition, the teacher of the group informed the children’s parents by e-mail and attached the information sheet and consent forms. The YRs were given 5 weeks to decide whether they wanted to participate, with one reminder e-mail to the parents. Parental consent was obtained in all cases. The ARs received an e-mail with detailed information about the study. They also signed an informed consent form before the interviews with them started.

### Data collection

The group interviews with the YRs took place during a meeting at the foundation and were conducted in two groups. We deliberately chose to perform group interviews rather than individual interviews to put the YRs at ease. We divided them into two smaller groups to make sure they all had a chance to answer the questions. One group consisted of 3 YRs and the other of 4 YRs. The group interviews lasted 30 min. The semi-structured interviews with the ARs took place at their workplaces. On average, the interviews lasted 60 min. A topic guide (Appendix 1) was set up by the research team and used to structure the interviews. To give the YRs an idea of what a research process involved, we showed them an approximately 2-min YouTube video (Appendix 1). In it the four research phases, design, data collection, analysis, and evaluation, were discussed.

The interviews were conducted by LP. She was introduced to the participants as researcher and had no prior relationship with the participants. All the interviews were audio recorded and transcribed verbatim by an external company. After each interview, field notes were made to retain noticeable moments. The transcripts were returned and checked by the participants.

### Data analysis

We analysed the transcripts using qualitative content analysis as described by Graneheim and Lundman [19]. According to this method a text always contains multiple meanings and some degree of interpretation always occurs when reading a text. The analysis involved the following steps: 1. We read the transcripts to gain an overall understanding of their contents. 2. We identified meaningful units. 3. We assigned a descriptive code to the meaningful units. 4. We divided the codes into subcategories, which we subsequently sorted into categories. We used the Atlas.ti computer program to categorize the meaningful units in a structured way. Data collection and analysis occurred concurrently so that we could constantly compare between the codes. Coding was an iterative process in that the categories were continuously updated to ensure they reflected the new data. The thematic analysis was performed by LP. During the course of the analysis, the research team met repeatedly to discuss the codes that had been identified.

### Ethical approval

The Medical Ethical Review Committee of University Medical Center Groningen concluded that this study fell outside the scope of the Dutch Medical Research Involving Human Subjects Act [20] (M19. 230,655 2019, April 25th). The privacy and personal information that had been collected during this study was adequately protected and only available to the research team as agreed in the consent forms. During the interviews, the children as well as the adult participants had the right to end their participation at any time. On account of the fact that in the Netherlands it is not allowed to reward children, the participants did not receive a financial reward for participating in this study.

## Results

### Sample

We approached 30 children through the *Jonge Onderzoekers Groningen* foundation. Seven children (Table 2), two girls and five boys, aged between 9 and 14 years, decided to participate. We did not record children’s reasons for not participating. We approached 16 adult researchers to participate in the semi-structured interviews, five of whom consented (Table 3). Despite several reminders, four researchers failed to respond and seven researchers decided against participating for lack of time. Altogether we included 12 participants in our study.

**Table 2:** Characteristics of the healthy children included

Characteristics	Young researchers
**Included**	7 out of 30
**Sex**	
Boys	5
Girls	2
**Age**	
9	1 (14)
11	1 (14)
12	3 (43)
14	2 (29)

**Table 3:** Characteristics of the adult researchers included

Characteristics	Adult researchers
**Included**	5 out of 16
**Sex**	
Men	4
Women	1
**Medical specialties**	
Paediatric oncology	1
Paediatric intensivist	1
Paediatric pulmonology	1
Paediatric metabolic diseases	1
General paediatrics	1

### Themes

The interviews consisted of six themes (Appendix 1). Below, we explain in detail the four themes that were discussed most extensively during the interviews: involving children in paediatric research, training children, training researchers, and rewarding children. Two themes were not discussed extensively and were therefore not included in this article.

### Involving children in paediatric research

YR: The YRs were unambiguously positive about involving children in paediatric research and agreed that children can be involved, and should be involved. They were surprised that to date, so few children had been involved in paediatric research at all. In their view, the added value of involving children is that they could raise different topics to those raised by the adults. They mentioned, for example, that in comparison to adults, children are more likely to think outside the box. Besides, children could add ideas that would make research more fun for children and easier to understand.

‘Children can complement adult ideas, because adult ideas are good, but if children supplement them, it might be great!’ (Boy, aged 12)

‘Children tend to have more creatives ideas, not always, but they tend to. They usually know stuff about their generation better than the ancient (*sic*) adult.’ (Boy, aged 12)

The YRs were convinced they could be involved in every phase of the research.

‘I think we can be involved in every step of the research; we can figure out what we want to study, we can think of how we want to study that, and we can also evaluate this process.’ (Girl, aged 14)

AR: The ARs thought it would be best to involve children in the research process from as early a stage as possible and, preferably, to include them in all stages of research. Besides, children should be included as part of the research team. The ARs also assumed that children could help improve research by including their perspective from the beginning, so that their suggestions could be included from the outset.

‘During the conceptual process, before research plans are made, it would be very nice to hear from children: what would you like to see researched? Children are open-minded, which could lead to funny, but very worthwhile proposals nevertheless.’ (Paediatrician, Hospital 1)

### Training children

YR: The YRs were clear about how they perceived the need for training. All seven were convinced that a short explanation about what is expected of them should suffice. They saw no added value in, for example, a weekly training session on ‘How to be involved in child-inclusive research’*.* The YRs compared doing research to their own school projects:

‘Actually, those phases are the same as for giving a talk at school, something children do all the time.’ (Girl, aged 14)

AR: The views of ARs on training children were varied. On the one hand, ARs thought training children would interfere with their open-mindedness. They stated that if you wanted to involve children you should give them the opportunity to talk freely, and without any inhibitions, about what was on their minds and, as AR, you should trust them. On the other hand, ARs did assume that children needed training. They thought that children might not have a clue about what is and what is not possible. They assumed that children needed some guidance in the same way ARs do.

‘Children do not have to know every rule, but they do need a framework to contain their ideas.’ (Researcher, Hospital 1)

### Training researchers

YR: The YRs were more differentiating about training for ARs. One YR thought that ARs did not need training to involve children in child-inclusive research because ARs already know how to do research and had once been children themselves. They could combine this knowledge to involve children in research:

‘Adults were children themselves once, so they know how to approach children in the right way.’ (Boy, aged 11)

Another YR concluded that researchers should have experience in working with children.

‘Training is not necessary: it depends on the experience of the adult in dealing with children. If not training may be recommendable.’ (Boy, aged 12)

AR: The ARs were positive about a brief training session on how to involve children in research. They offered suggestions about the content and the design of such training for researchers. The ARs saw no added value in training about how to communicate with children because it was something they did every day. According to the ARs, training could be limited to a one-day session dealing with specific guidelines on how to involve children in research.

‘You cannot simply involve children in research, you need to have some background information (about child-inclusive research – LP) given during a one-day training session.’ (Paediatric intensivist, Hospital 1)

‘How can I involve a child as co-researcher in a way he or she really becomes part of the team?’ (Paediatrician, Hospital 2)

### Rewarding children

YR: The YRs had different opinions about the need for a financial reward when they were involved in paediatric research. As 1 YR mentioned:

‘I do not need a financial reward, I have a lot of stuff so I do not need anything extra, just a ‘Good job’ or a ‘Thank you for your time’ would be enough for me.’ (Girl, aged 14)

Another YR agreed that a financial reward was not necessarily but he did feel that it might provide additional motivation.

‘I do not need a financial reward, but yeah, I think that it would be the best way to encourage children and they will be more likely to do a good job.’ (Boy, aged12)

Some YRs then continued to discuss what amount would be a fair financial reward. They considered that the reward ought to be modest but had difficulties defining ‘modest’.

‘Ten euros is way too much, two euros should be better, or a small chocolate bar, or sweets, or something like that.’ (Girl, aged 14)

Generally, the interviews revealed that YRs felt that recognition and a modest financial reward would suffice.

AR: The ARs were clear about not rewarding children financially. They all thought that children should not be paid for their involvement. The reason for children wanting to be involved in research should not depend on whether a financial reward was forthcoming. An important point made by one of the ARs was that children should be reimbursed for their travel expenses and any other costs they may have incurred on account of the research.

‘I do not think you should reward the children financially. If I were to include children in research, I would only include children who are intrinsically motivated.’ (Paediatrician, Hospital 2)

‘It is nice to give children something in return, but it should never be the reason for the child to decide to be involved in research; they should become involved because they think it is interesting.’ (Researcher, Hospital 1)

## Discussion

In this exploratory study on the attitudes of YRs and ARs towards the challenges frequently encountered during child-inclusive research, we noticed that the children’s answers were reflexive, thoughtful, and critical. Most children were able to argue their motivations well. They asked each other critical questions in case they disagreed on something. Both the YRs and the ARs thought it would be best to involve children in research from as early a stage as possible; preferably in all stages of research. The YRs thought that they did not need prior training because doing research resembled projects they do for school. Opinions differed amongst the ARs. Some thought training YRs would interfere with their open-mindedness, while others thought YRs should be told what to expect during the research project they were to be involved in. The YRs assumed that the ARs did not need training in how to involve children in medical research, while the ARs thought training researchers would have added value. Regarding the issue of a financial reward, the YRs felt that recognition, or a modest financial reward, would suffice. The ARs had reservations about rewarding children; they feared children might no longer be intrinsically motivated.

### Involving children in paediatric research

The views of both ARs and YRs in this study correspond to the literature on the possibility of involving children in child-inclusive research [17, 18, 21]. Bradburry-Jones and colleagues argued that children should not have to prove capacity. Researchers should assume that children are competent to form their own opinions [18]. The list of recommendations for researchers issued by the National Institute of Health Research (NIHR) INVOLVE group states ‘do not make assumptions about what we are interested in or capable of – ask us’ [22].

As child-inclusive research is becoming more and more common in medical research, it could be viewed as a necessary part of an application for funding [8]. Sometimes child-inclusive research is experienced as a ‘tick box’ exercise [23]. Our study showed that the ARs who had no prior experience with or previous interest in child-inclusive research, thought it best to involve children from the outset. This was important because in this way the children’s input could be considered from the first stage of the research. An example in which patients were involved form the beginning is the Priority Setting Partnerships facilitated by the James Lind Alliance. These partnerships enable clinicians, patients, and carers to work together to identify and prioritise evidence uncertainties that could be answered by research. The attitudes of researchers - no longer considering child-inclusive research as an obligation but acknowledging its added value to research - might be a significant step towards the meaningful involvement of children.

### Training children

Regarding the need to train children, our results differed to those found in the literature. The literature suggests children should be supported with adequate training, although training is interpreted in different ways [18, 24]. Kellet argued that training children to be involved in child-inclusive research is clearly necessary. For that purpose, she developed an interactive training program. It teaches children about the nature of research, the different approaches to research, about research ethics, and about how to frame a research question. In addition, the program teaches children about data collecting techniques and data analysis [25]. Contrarily, Gaillard and colleagues stated that children can be trained in research through participation in young person advisory groups. Children can assist each other to learn about research and share their experiences [12]. In the study of Luchtenberg and colleagues, children were involved in analyzing qualitative data. To avoid shaping children with the ideas of a qualitative researcher, they were not trained deliberately. The children received a brief interactive introduction about pediatric research. Most of the children said that before the actual analysis started, they only understood their role a little bit. As the research process proceeded, their role became clearer to them and the children grew more confident as time went by. Luchtenberg and colleagues hypothesized that limited training does not have a negative impact on the results [26]. This might indicate that children do not need to be trained on how to be involved in child-inclusive research. A brief introduction suffices. This idea is in line with the results of our study. The YRs indicated they did not see any added value in, for example, weekly training sessions, because in their opinion they had already picked up the necessary knowledge at school. This attitude was also described by Gaillard and colleagues who state that children learn about research during other activities [12]. Dudley and colleagues interviewed children who had prior experience in child-inclusive research about the need for training children prior to involving them. Children included in that study mentioned that they would benefit from training on how child-inclusive research works, on how to have the confidence to speak up, and about what is expected from them [27]. These results indicate that children do not need substantive training on how to be involved in child-inclusive research, as argued by Kellet [25]. What they do need is information about what it is like to be involved in research.

The YRs in our study were recruited from a foundation that offers them the opportunity to develop their interests in technology and sciences. The YRs all had a specific interest in science. They probably also learnt a great deal about actually performing research during their lessons at the foundation, which made training less relevant. Children with no such interest or experience in research would probably benefit from training.

### Training researchers

The YRs assumed that adult researchers did not need training on how to involve children in research if they already worked with children on a daily basis. This corresponds partly to the attitudes of the ARs. They stated that they saw no added value in being trained in how to communicate with children, because that was something they were used to. The ARs did indicate that they needed a brief training on how to involve children in research. This is also reflected in a survey by Winch and colleagues. They identified the challenges hospital doctors encounter when they want to involve children in research and their thoughts on how this issue should be addressed [28]. More guidance, training, and information should be available on overcoming the inequality in the relationships between children and adult researchers when involving children in medical research [28, 29]. The literature offers scant information on the attitudes of children regarding the need for training for adult researchers on involving children. The INVOLVE “tips for researchers”, which is composed together with children, also does not include training for researchers. It is not clear whether they did not think of adult training at all, or whether it was not deemed important. In the Netherlands, ZonMw (the Dutch organization for health research and healthcare innovation) provides master classes with tips and tricks for researchers who want to involve children in research. It is worth noting that in our study, the YRs themselves started discussing the need to train researchers when we talked about training children.

### Rewarding children

Reciprocity and reward are important factors in child-inclusive paediatric research [13, 22, 30]. The YRs suggested that recognition and a modest financial reward was sufficient. This is in line with the list INVOLVE published with points to consider when involving children in research. They stated that children should receive an appropriate reward and recognition for their contributions, to demonstrate that their time, commitment, and expertise are valued [30]. The list was used in two studies to determine what reward children should receive in child-inclusive research [13, 14]. Mitchel and colleagues recommended giving children ‘Thank You’ certificates, vouchers, and events organized in partnership with the PPI group [24]. Results from our YR interviews were consistent with the list published by INVOLVE. Moreover, they endorsed the statement by Mitchell and colleagues to devise a form of reward in consultation with the children.

A noticeable finding in our study were the views held by the ARs regarding the need to reward the children. The ARs thought it was a thoughtful gesture to compensate the children, but they were not in favour of a financial reward. The ARs stressed the importance of children being recognised, but above all that the reason for becoming involved should not depend on a financial reward. To our knowledge, little is known about the attitudes of researchers concerning this matter. Modi wrote about what adults think of rewarding children who are involved in research. Some adults think it might pose danger, and that it goes against the principle of autonomous decision-making if a child feels tempted because of a financial reward [31]. Furthermore, the YR interviews showed that the children themselves indicated that they did not need a financial reward to be involved. Nevertheless, recognition of their expertise and the time invested would be appreciated. This corresponds to the results of Forsyth and colleagues [13].

### Limitations and future research

A limitation of our exploratory study was that the number of YRs and ARs included was rather small. The children we included had some experience in research in order to compare their perspectives with those of adult researchers. The YRs in this study may have influenced the results regarding the need for training before involvement in research. Seeing that children with disabilities are sometimes involved in child-inclusive research as well [7], it would be interesting to compare their attitudes to the attitudes of the YRs included in this study. Larger studies too should focus on the involvement of both paediatric patients and healthy children in order to examine the difference between these two groups. The outcomes could be used to determine whether their expertise differs in the same way as in adult involvement, as described by McCoy [2].

## Conclusion

Our study indicated that young and adult researchers have clear attitudes towards the challenges encountered during child-inclusive research. Young researchers could help adult researchers to find solutions to these challenges, even if they have no prior experience in child-inclusive research. Adult researchers who acknowledge the importance of child-inclusive research represent a significant step towards meaningful involvement of children. Our results imply that children could be involved in the decision-making process concerning the challenges encountered in child-inclusive research.

## Data Availability

The datasets used and analysed during the current study are available from the corresponding author on reasonable request.

## Appendix 1

### Topic guide

#### Introduction

- Introduce myself
- Aim of the research
- Summary of this interview

With children show: https://www.youtube.com/watch?v=Uz8T5-BG-R4&t=49s

**Topic 1: Familiarity with patient and public involvement (PPI)**

- What do you know about PPI?
- Where did you hear about PPI?
- Did you ever think about involving children in your research?
- Why did you not involve children in research before?
- Importance of PPI

**Topic 2: involvement of children in research**

- Possible to involve children? Why?
- In what phase of research should children be involved?
- What roles could apply to children?
- In what why should children be involved?
- In particular circumstances?

**Topic 3: Training children**

- Do children need training to be involved in research?
- What should the training focus on?
- How long should the training last?
- Should every child receive this training

**Topic 4: Training researchers**

- Do researchers need training to involve children in research?
- What should the training focus on?
- How long should the training last
- Should every researcher receive this training

**Topic 5: Rewarding children**

- Do children need a reward to be involved in research?
- What is an appropriate way of rewarding children that are involved?

**Topic 6: Disadvantages/advantages of involvement of children**

- What are advantages of involving children in research?
- What are disadvantages of involving children in research?

## Abbreviations

AR: Adult researcher
PPI: Patient and Public Involvement
UMCG: University Medical Center Groningen
UNCRC: United Nations Convention on the Rights of the Child
YR: Young researcher
